# Effects of Ambient Oxygen Concentration on Microstructural Evolution and Mechanical Properties of Wire Arc Additively Manufactured Ti-6Al-4V Thin-Walled Components

**DOI:** 10.3390/ma19112347

**Published:** 2026-06-02

**Authors:** Shuo Meng, Zonglin Zhao, Hongwei Ji, Guangkuo Qin, Yefei Zhou, Weidong Ma, Xiaolei Xing

**Affiliations:** 1 College of Mechanical Engineering, Yanshan University, Qinhuangdao 066004, China; mengshuo_2020@163.com (S.M.); 17722490544@163.com (Z.Z.); m19833356670@163.com (H.J.); 17849710169@163.com (G.Q.); yfzhou@ysu.edu.cn (Y.Z.); 2 China Electronics Technology Group Corporation No. 46 Research Institute, Tianjin 300220, China; 3 State Key Laboratory of Metastable Materials Science & Technology, Yanshan University, Qinhuangdao 066004, China; 4 School of Mechanical and Electrical Engineering, Handan University, Handan 056005, China

**Keywords:** Ti-6Al-4V titanium alloy, wire arc additive manufacturing, ambient oxygen concentration, surface oxidation, tensile properties

## Abstract

Ti-6Al-4V thin-walled specimens were fabricated by gas tungsten arc welding-based wire arc additive manufacturing under controlled oxygen concentrations of 1, 500 and 1000 ppm, with ambient air used as a severe oxygen-exposure reference. The effects of oxygen concentration on oxygen uptake, microstructure, oxidation behavior and mechanical properties were investigated. Within the controlled range, the internal oxygen content increased from 0.07 to 0.15 wt.%, remaining below the ASTM B381-2013 limit. These specimens retained sound interlayer bonding and were mainly composed of α-Ti with a small amount of β-Ti, without detectable crystalline TiO_2_ by X-ray diffraction. Controlled oxygen uptake refined the α lamellae and increased deformation resistance through interstitial solid-solution strengthening, increasing hardness from approximately 320 HV to 330–350 HV and tensile strength from 880 to 940 MPa, while reducing elongation from 11.5% to 9.5%. In contrast, the ambient-air specimen reached an oxygen content of 0.36 wt.%, developed an approximately 90 μm oxidation-affected layer and showed TiO_2_-related oxides, α-colony aggregation and interface weakening. Its tensile strength and elongation decreased sharply to 295 MPa and 1.9%, respectively. These results indicate that atmosphere control in WAAM Ti-6Al-4V should prevent the transition from controlled oxygen strengthening to excessive oxygen-induced embrittlement.

## 1. Introduction

Ti-6Al-4V titanium alloy possesses a high specific strength, good corrosion resistance and favorable high-temperature service capability, and has therefore been widely used in aerospace, marine engineering, biomedical applications and high-end equipment manufacturing [1,2,3]. For large titanium alloy frames, stiffeners, integral skeletons and complex load-bearing structures, conventional subtractive manufacturing is often limited by low material utilization, long processing cycles and high manufacturing costs [4,5]. Wire arc additive manufacturing (WAAM) uses an electric arc as the heat source and metal wire as the feedstock to achieve near-net shaping of large metallic components through layer-by-layer melting and deposition. Owing to its high deposition efficiency, low equipment cost and high material utilization, WAAM is considered one of the important processing routes for fabricating large titanium alloy components [6,7,8].

However, Ti-6Al-4V titanium alloy is highly sensitive to atmospheric impurities such as oxygen, nitrogen and water vapor during high-temperature melting and solidification [9,10]. Oxygen is a typical interstitial element in α-Ti. A small amount of oxygen entering the titanium matrix can induce lattice distortion and increase the resistance to dislocation glide, thereby producing a solid-solution strengthening effect [11,12,13]. Therefore, within a certain range, an increase in oxygen content generally improves the hardness and strength of titanium alloys. Nevertheless, oxygen has a pronounced dual effect on Ti-6Al-4V. When the oxygen content is excessive, it significantly reduces ductility and the capacity for coordinated plastic deformation, promotes brittle fracture behavior, and may lead to the formation of oxide particles or oxide layers in local regions, thereby weakening interfacial bonding and microstructural continuity [14,15,16]. This issue becomes more complex for WAAM Ti-6Al-4V. During layer-by-layer fabrication, the deposited layers repeatedly undergo high-temperature exposure, surface oxidation, interlayer reheating and local remelting. Oxygen may not only enter the deposited layers through surface oxidation, but may also be further entrained into the molten pool during subsequent remelting and accumulate inside the matrix [17]. Therefore, the oxygen concentration in the forming atmosphere can simultaneously affect surface oxidation, internal oxygen uptake, microstructural evolution and mechanical properties of WAAM Ti-6Al-4V.

Current studies on WAAM Ti-6Al-4V have mainly focused on heat input, deposition path, interlayer temperature, grain morphology, residual stress and heat treatment [18,19]. Previous studies have shown that WAAM Ti-6Al-4V usually exhibits coarse prior β columnar grains, lamellar α microstructures and pronounced anisotropic mechanical behavior [20,21]. To improve its microstructure and properties, methods such as interlayer rolling, external magnetic fields, ultrasonic assistance, heat treatment and process parameter optimization have been proposed [22,23]. In contrast, it remains insufficiently understood how the oxygen concentration in the forming atmosphere affects the relationships among internal oxygen uptake, surface oxide layer formation, α-lamellar structure, phase constitution and tensile fracture behavior of WAAM Ti-6Al-4V [24]. In particular, it is necessary to clarify the critical transition from controlled oxygen uptake to harmful oxygen contamination under the repeated thermal cycles and interlayer remelting conditions of WAAM, together with the corresponding microstructure–property mechanisms. This issue is not only related to the mechanical reliability of additively manufactured Ti-6Al-4V components, but also to the balance between shielding-atmosphere purification cost and quality-control requirements in practical manufacturing [10]. Therefore, clarifying the microstructural and mechanical responses of WAAM Ti-6Al-4V under different ambient oxygen concentrations is of great significance for establishing a reasonable atmosphere-control window.

In this study, Ti-6Al-4V thin-walled specimens were fabricated by GTAW-WAAM under controlled oxygen concentrations of 1 ppm, 500 ppm and 1000 ppm, with an ambient-air condition introduced as a severe oxygen-exposure reference. Although the role of oxygen as an α stabilizer and interstitial strengthening element in titanium alloys is well known, its coupled influence on oxygen uptake, surface oxidation, α-lamellar evolution and tensile fracture behavior under the repeated thermal exposure and interlayer remelting conditions of GTAW-WAAM remains insufficiently clarified. Different from previous studies that mainly emphasized oxygen contamination, alpha-case formation or shielding effectiveness under insufficient protection, this work aims to distinguish controlled oxygen uptake from severe oxygen contamination in GTAW-WAAM Ti-6Al-4V. The scientific advancement is to clarify how limited oxygen uptake regulates α-lamellar morphology and mechanical response without destroying structural continuity, and how ambient-air exposure changes this response into oxidation-related microstructural degradation and brittle failure. By correlating oxygen uptake with surface oxidation, α-lamellar morphology, phase constitution, hardness distribution, tensile response and fracture behavior, the present work focuses on systematically evaluating the controlled oxygen range up to 1000 ppm in GTAW-WAAM Ti-6Al-4V and identifying the transition from controlled oxygen solid-solution strengthening to excessive oxygen-contamination-induced embrittlement. By establishing the relationships among ambient oxygen concentration, internal oxygen content, microstructural evolution and mechanical response, this study provides experimental evidence for atmosphere control and engineering application of WAAM Ti-6Al-4V.

## 2. Materials and Methods

### 2.1. Materials and Experimental Setup

Ti-6Al-4V titanium alloy wire with a diameter of 1.0 mm was used as the deposition feedstock and was continuously supplied to the molten pool by a wire-feeding unit. The substrate was also made of Ti-6Al-4V titanium alloy, with dimensions of 120 mm × 50 mm × 5 mm. Before deposition, the substrate surface was mechanically ground to remove the oxide layer and then cleaned with ethanol to remove surface oil and impurities.

The wire arc additive manufacturing system consisted of a gas tungsten arc welding unit, a wire-feeding unit, a three-axis motion control unit, a water-cooling system and an atmosphere purification system. The deposition process was carried out in a modified sealed glove box. High-purity argon was introduced into the glove box, and a circulating purification system was used to reduce the oxygen and water vapor contents inside the chamber. Under controlled atmosphere conditions, the oxygen concentration in the glove box was set to 1 ppm, 500 ppm and 1000 ppm, respectively. To compare the microstructural and property changes under extreme oxygen exposure, another group of specimens was fabricated in ambient air. It should be noted that the air-fabricated specimen was used as a non-controlled high-oxygen exposure control to characterize microstructural deterioration and mechanical property degradation under severe oxygen contamination, rather than as a strictly quantitative oxygen concentration point for linear comparison.

### 2.2. Fabrication of Thin-Walled Specimens

**Figure 1:**
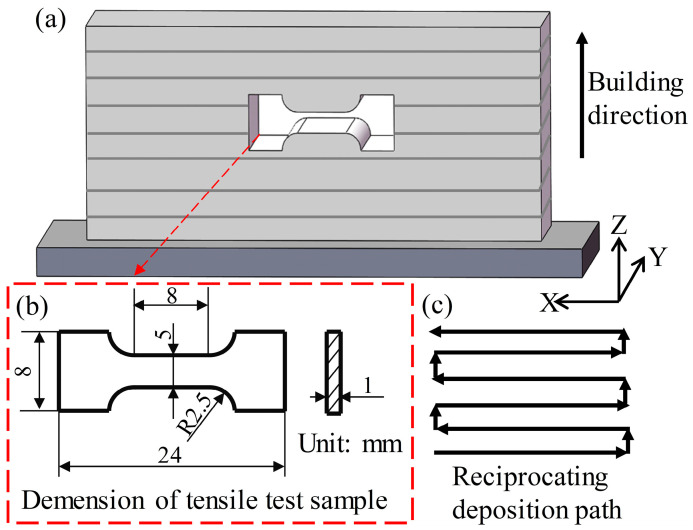
Schematic illustration of the WAAM Ti-6Al-4V thin-walled specimen, tensile specimen geometry and deposition path. (**a**) Sampling position of the tensile specimen in the deposited thin wall, (**b**) dimensions of the tensile test sample, and (**c**) reciprocating deposition path.

Ti-6Al-4V thin-walled specimens were fabricated using a reciprocating deposition path. The schematic illustration of the deposited thin-walled specimen, tensile specimen geometry and reciprocating deposition path is shown in Figure 1. The tensile specimens were extracted from the deposited wall, and their geometry and dimensions are given in Figure 1b. The reciprocating deposition strategy used for layer-by-layer fabrication is shown in Figure 1c. The four groups of specimens were denoted as A, B, C and D, corresponding to ambient oxygen concentrations of 1 ppm, 500 ppm, 1000 ppm and ambient air, respectively. The same deposition parameters were used for all specimens to eliminate the effects of differences in heat input, wire feeding speed and deposition path on the microstructure and properties. The deposition current was 160 A, the arc voltage was 20 V, the travel speed was 200 mm/min, the wire feeding speed was 1300 mm/min, the argon flow rate was 8 L/min, and the interlayer dwell time was 180 s. The designed height, length and wall thickness of the thin-walled specimens were approximately 24 mm, 90 mm and 9.5 mm, respectively. The effective linear heat input, calculated using E = ηUI/v with a thermal efficiency η of 0.75, was approximately 720 J/mm.

### 2.3. Characterization Methods

After deposition, metallographic specimens were sectioned along the cross-sectional direction of the thin-walled specimens. The specimens were ground sequentially using SiC abrasive papers with different grit sizes and then mechanically polished with diamond polishing suspension. The polished specimens were etched using Kroll’s reagent, consisting of 2 vol.% HF, 6 vol.% HNO_3_ (Qinhuangdao Zhisheng Trading Co., Ltd., Qinhuangdao, China) and deionized water, for 15–20 s. The macrostructure was observed using an Axiovert 200 MAT optical microscope (Carl Zeiss Microscopy GmbH, Göttingen, Germany), while the microstructure and fracture morphology were examined using a Hitachi S4800 field-emission scanning electron microscope (Hitachi High-Technologies Corporation, Tokyo, Japan).

The phase constitution of the specimens fabricated under different oxygen concentrations was analyzed by X-ray diffraction (Rigaku Corporation, Akishima-shi, Tokyo, Japan). The accelerating voltage and current were 40 kV and 30 mA, respectively. The scanning range was 30–80°, with a step size of 0.02°. For the oxide diffraction peaks observed in the specimen fabricated in ambient air, the phase identification was further evaluated in combination with SEM observations.

The oxygen contents of the deposited specimens were measured by the inert gas fusion method using an oxygen/nitrogen/hydrogen analyzer (LECO Corporation, St. Joseph, MI, USA). For each condition, representative bulk samples were cut from the middle-height region of the deposited wall, and the sampling position was kept consistent among all specimens. Before measurement, the outer oxidized surface and visibly contaminated regions were mechanically removed. Therefore, the reported oxygen contents represent the bulk oxygen uptake of comparable regions in the deposited specimens under different atmosphere conditions, rather than position-resolved oxygen distributions along the building direction or from the surface to the interior.

Microhardness tests were conducted on the polished cross-sections of the thin-walled specimens using a Vickers microhardness tester (Qness GmbH, Golling, Austria) under a load of 200 g and a dwell time of 10 s. To evaluate the hardness variation along the building direction, the indentations were arranged along the vertical direction of the cross-section with a spacing of 2 mm between adjacent positions. To further characterize the hardness gradient from the surface to the interior, Vickers microhardness measurements were performed along the depth direction, with a measurement interval of 10 μm. For each condition, at least three repeated measurements were performed, and the results are reported as mean values with standard deviations. During measurement, the indentation positions were selected away from visible pores, cracks, oxide-related features and interfacial discontinuities to reduce the influence of local defects on the hardness results.

Tensile specimens were sectioned from the thin-walled specimens, and three specimens were prepared for each group. Tensile tests were conducted at room temperature with a displacement loading rate of 1 mm/min. After testing, the tensile strength and elongation after fracture were measured, and the fracture morphologies were observed using scanning electron microscopy to analyze the fracture mechanisms under different oxygen concentration conditions.

## 3. Results and Discussion

### 3.1. Specimen Characteristics and Composition

**Figure 2:**
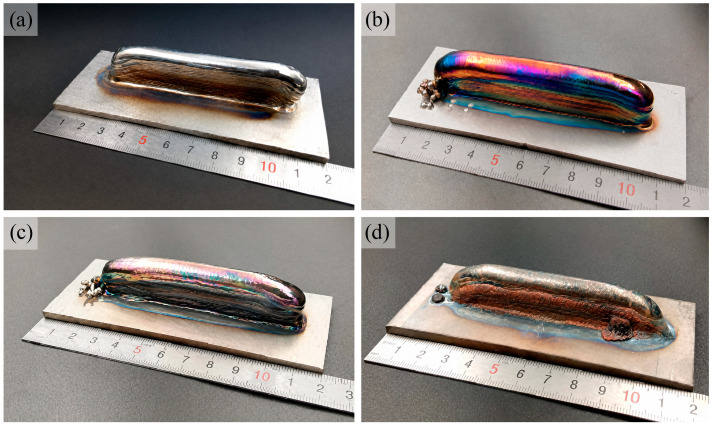
Macroscopic surface morphologies of GTAW-WAAM Ti-6Al-4V thin-walled specimens fabricated under different ambient oxygen concentrations. (**a**) 1 ppm, (**b**) 500 ppm, (**c**) 1000 ppm, and (**d**) ambient air.

Figure 2 shows the macroscopic morphologies of the WAAM-fabricated Ti-6Al-4V thin-walled specimens under different ambient oxygen concentrations. The surface color of the specimens changed markedly with increasing oxygen concentration in the forming atmosphere. The specimen fabricated at 1 ppm exhibited a silvery-white surface (Figure 2a), indicating a relatively low degree of surface oxidation. The specimen fabricated at 500 ppm showed a yellow surface with locally blue regions (Figure 2b). When the oxygen concentration increased to 1000 ppm, the specimen surface further changed to a blue-gray color with local purple regions (Figure 2c). In contrast, the specimen fabricated in ambient air exhibited a gray-brown surface (Figure 2d), indicating a significantly enhanced degree of surface oxidation.

Ti-6Al-4V titanium alloy has a strong affinity for oxygen during high-temperature deposition [25]. When the deposited layer is at elevated temperature, oxygen in the surrounding atmosphere readily reacts with titanium and forms an oxide film on the specimen surface. Under different oxygen concentration conditions, the amount of oxygen absorbed by the specimen surface varies, resulting in differences in the thickness of the oxide film formed. Owing to the interference effect of a thin oxide film on incident and reflected light, oxide films with different thicknesses can enhance or weaken light of different wavelengths, thereby causing different surface colors [26]. Therefore, changes in surface color can serve as an intuitive indicator of the degree of surface oxidation in titanium alloys. As the ambient oxygen concentration increased from 1 ppm to 1000 ppm, the surface color of the specimens gradually deepened, suggesting enhanced surface oxidation. This interpretation is further supported by the subsequent cross-sectional SEM analysis of the oxidation-affected layer. The gray-brown surface of the specimen fabricated in ambient air indicates that its surface oxidation reaction was significantly more severe than that of the specimens fabricated under controlled low-oxygen atmospheres.

Table 1 lists the representative bulk chemical compositions of the Ti-6Al-4V specimens fabricated under different atmosphere conditions. The representative bulk oxygen contents of specimens A, B, C and D were 0.07 wt.%, 0.10 wt.%, 0.15 wt.% and 0.36 wt.%, respectively. The oxygen contents of the specimens fabricated at 1 ppm, 500 ppm and 1000 ppm remained below the upper limit of 0.20 wt.% specified by ASTM B381-2013 [27], whereas the specimen fabricated in ambient air clearly exceeded this limit. In contrast, the Al and V contents varied only slightly, indicating that the atmosphere condition mainly affected oxygen uptake rather than the contents of the major alloying elements.

The increase in internal oxygen content is associated with repeated high-temperature exposure during WAAM. Surface oxidation can occur on the previously deposited layer, and part of the oxidation-affected surface may be remelted or entrained into the molten pool during subsequent deposition. Therefore, the measured oxygen content depends not only on the nominal oxygen concentration in the atmosphere, but also on the repeated oxidation, remelting and thermal exposure history of the deposited wall.

It should be noted that ambient air is fundamentally different from the controlled oxygen atmospheres of 1 ppm, 500 ppm and 1000 ppm. The latter three conditions represent controlled oxygen concentration environments and can be used to analyze the progressive effects of ambient oxygen concentration on internal oxygen uptake, microstructural evolution and mechanical properties. By contrast, ambient air represents a non-controlled high-oxygen exposure condition, in which the oxygen level is much higher than that in the controlled atmospheres and may also be accompanied by impurities such as nitrogen and water vapor. Therefore, the air-fabricated specimen is more appropriately regarded as a failure control under severe oxygen contamination, rather than as a quantitative point in a controlled oxygen concentration gradient.

These results indicate that the specimens fabricated under 1–1000 ppm oxygen atmospheres represent a controlled oxygen-uptake regime, whereas the ambient-air specimen represents a severe oxygen-contamination condition. This distinction provides the basis for the following analysis of microstructure, hardness and tensile behavior.

### 3.2. Microstructural Evolution Under Different Oxygen Concentration Conditions

**Figure 3:**
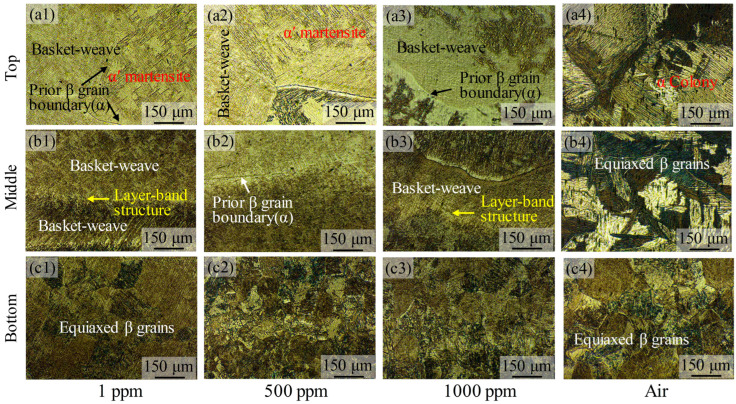
Low-magnification cross-sectional microstructures of WAAM Ti-6Al-4V thin-walled specimens fabricated under different ambient oxygen concentrations. (**a1**) Top region of the 1 ppm specimen, (**a2**) top region of the 500 ppm specimen, (**a3**) top region of the 1000 ppm specimen, (**a4**) top region of the ambient-air specimen, (**b1**) middle region of the 1 ppm specimen, (**b2**) middle region of the 500 ppm specimen, (**b3**) middle region of the 1000 ppm specimen, (**b4**) middle region of the ambient-air specimen, (**c1**) bottom region of the 1 ppm specimen, (**c2**) bottom region of the 500 ppm specimen, (**c3**) bottom region of the 1000 ppm specimen, and (**c4**) bottom region of the ambient-air specimen.

Figure 3 shows the optical microstructures of the top, middle and bottom regions of the WAAM Ti-6Al-4V thin-walled specimens fabricated under different ambient oxygen concentrations. For the specimens fabricated under controlled oxygen concentrations of 1 ppm, 500 ppm and 1000 ppm, continuous metallurgical bonding between adjacent deposited layers can be observed, and no obvious lack-of-fusion defects, cracks or large oxide inclusions are detected. This indicates that, under the present GTAW-WAAM parameters, increasing the oxygen concentration from 1 ppm to 1000 ppm does not destroy the overall forming quality or interlayer bonding of the deposited walls.

For the 1 ppm specimen, the top and middle regions mainly consist of a typical basket-weave microstructure formed within prior-β grains, as shown in Figure 3(a1,b1). Grain-boundary α can also be observed along the prior-β grain boundaries in the top region, while local acicular α′ martensite or fine α features appear in Figure 3(a1). This morphology suggests that the newly deposited upper layers experienced a relatively high cooling rate after solidification. In the middle region, a distinct layer-band structure is visible in Figure 3(b1), which is associated with the repeated reheating and thermal cycling caused by subsequent layer deposition. In contrast, the bottom region exhibits more evident equiaxed prior-β grain features, as shown in Figure 3(c1), reflecting the combined effects of substrate heat extraction, repeated thermal exposure and partial thermal stabilization during multilayer deposition.

When the ambient oxygen concentration increases to 500 ppm and 1000 ppm, the overall microstructural framework remains similar to that of the 1 ppm specimen. Basket-weave microstructures are still dominant in the top regions, as shown in Figure 3(a2,a3), and prior-β grain boundaries decorated by grain-boundary α can still be identified. In the middle regions, layer-band structures are observed in Figure 3(b2,b3), indicating that the characteristic thermal-cycle-induced microstructure of WAAM Ti-6Al-4V is not fundamentally changed by controlled oxygen uptake. However, compared with the 1 ppm specimen, the local α morphology in the 500 ppm and 1000 ppm specimens appears finer and more densely distributed, suggesting that oxygen uptake within this controlled range mainly affects the β → α transformation and α-lamellar morphology, rather than causing macroscopic microstructural damage.

The microstructure of the specimen fabricated in ambient air is markedly different from those fabricated under controlled oxygen concentrations. In the top region, coarse α colonies with relatively concentrated lamellar orientations are observed in Figure 3(a4), and the basket-weave morphology becomes less uniform. In the middle and bottom regions, equiaxed prior-β grains are more prominent, as shown in Figure 3(b4,c4), while the layer-band characteristics become weakened. These features indicate that ambient-air exposure changes the microstructural evolution from limited oxygen-assisted lamellar regulation to severe oxygen-contamination-induced structural instability. The formation of coarse α colonies and the weakening of the layered microstructural features suggest that excessive oxygen exposure reduces the continuity of the thermal-cycle-induced microstructure and promotes local microstructural inhomogeneity.

Figure 3 demonstrates two distinct microstructural regimes. Under controlled oxygen concentrations from 1 ppm to 1000 ppm, the WAAM Ti-6Al-4V specimens retain continuous interlayer bonding, basket-weave microstructures and layer-band features, while oxygen mainly modifies the local α morphology. Under ambient-air exposure, however, excessive oxygen contamination promotes α-colony aggregation, weakens the layer-band structure and leads to a more heterogeneous prior-β grain morphology. This difference provides an important microstructural basis for understanding the subsequent changes in phase constitution, hardness distribution and tensile behavior.

**Figure 4:**
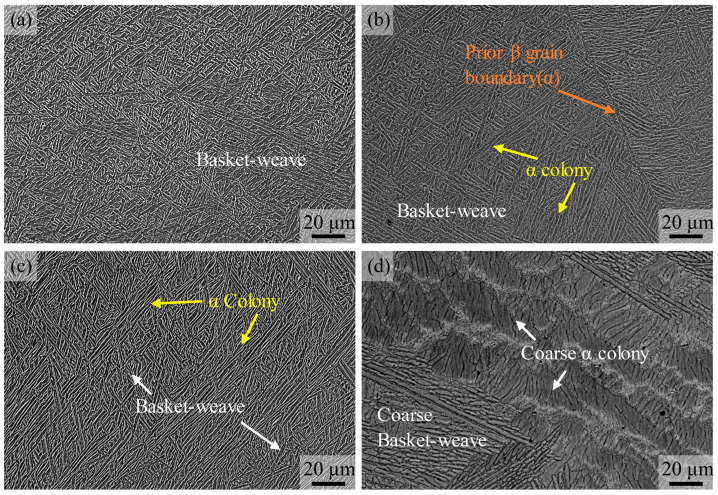
High-magnification SEM microstructures of WAAM Ti-6Al-4V specimens fabricated under different ambient oxygen concentrations. (**a**) 1 ppm, (**b**) 500 ppm, (**c**) 1000 ppm, and (**d**) ambient air.

Figure 4 shows the high-magnification SEM microstructures of the specimens fabricated under different ambient oxygen concentrations. It can be seen that the specimens fabricated under controlled oxygen concentrations were mainly composed of lamellar α phase and a small amount of retained β phase, which is the typical microstructure formed in WAAM Ti-6Al-4V under relatively high heat input and repeated thermal cycling conditions. At an oxygen concentration of 1 ppm, a relatively uniform basket-weave microstructure is observed, in which fine α lamellae intersect with each other within the prior β grains (Figure 4a). This interlaced morphology indicates that the β → α transformation occurred along multiple crystallographic orientations during cooling, without forming a strongly oriented colony structure. When the ambient oxygen concentration increased to 500 ppm, the basket-weave morphology was still retained, but local α colonies became more distinguishable (Figure 4b). The marked prior β grain boundary indicates that the original β-grain framework was partly preserved after the β → α transformation. Compared with the 1 ppm specimen, the α lamellae in the 500 ppm specimen appear finer and more directionally arranged in local regions, suggesting that controlled oxygen uptake modified the growth behavior of α lamellae without destroying the overall basket-weave framework. For the 1000 ppm specimen, α colonies became more evident, and the α lamellae within individual colonies showed a more parallel and elongated morphology (Figure 4c). Nevertheless, basket-weave regions were still present between adjacent colonies, indicating that the microstructure had not transformed into a fully coarse colony-dominated structure. This suggests that oxygen uptake within the controlled range mainly promoted α-lamellar refinement and local colony development, rather than causing severe microstructural degradation. In contrast, the specimen fabricated in ambient air exhibited a clearly different microstructure, characterized by coarse α colonies and coarse basket-weave regions (Figure 4d). The α lamellae showed increased spacing and stronger orientation concentration, reflecting a loss of microstructural uniformity under severe oxygen exposure. Therefore, the high-magnification SEM observations further confirm that the specimens fabricated at 1–1000 ppm remained in a controlled oxygen-regulated regime, whereas ambient-air exposure promoted local lamellar coarsening and α-colony aggregation.

**Figure 5:**
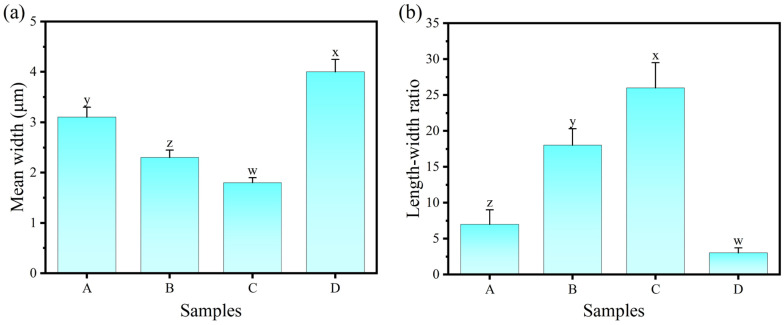
Statistical results of α-lamellar width and length-to-width ratio of WAAM Ti-6Al-4V specimens fabricated under different ambient oxygen concentrations. (**a**) Mean α-lamellar width; (**b**) length-to-width ratio. Samples A, B, C and D correspond to 1 ppm, 500 ppm, 1000 ppm and ambient air, respectively. Different letters indicate statistically significant differences among the specimens (*p* < 0.05).

The α-lamellar width and aspect ratio were statistically measured from high-magnification SEM images using ImageJ software (version 1.54). The statistical results of α-lamellar width and length–width ratio in Figure 5 further confirm the oxygen-dependent change in α-lamellar morphology. Within the controlled oxygen concentration range from 1 ppm to 1000 ppm, the α-lamellar width decreased, whereas the length–width ratio increased with increasing ambient oxygen concentration. This indicates that controlled oxygen uptake promoted α-lamellar refinement and elongation. This behavior may be associated with the role of oxygen as an α-stabilizing interstitial solute, which can increase lattice distortion and retard the lateral growth of α lamellae during the β → α transformation. However, the ambient-air specimen deviated from this trend and exhibited coarser α lamellae with a lower length–width ratio. This indicates that excessive oxygen exposure no longer contributed to beneficial lamellar regulation, but instead induced microstructural coarsening and inhomogeneity. Combined with the TiO_2_-related XRD peaks and the much thicker oxidation-affected layer observed in the ambient-air specimen, the lamellar coarsening under ambient-air exposure is more reasonably associated with severe oxygen contamination, local oxygen enrichment and oxide-related microstructural degradation.

This lamellar refinement can be understood from the effect of oxygen on the β → α phase transformation. Oxygen is a typical interstitial solute in α-Ti and also acts as an α-phase stabilizer. When a small amount of oxygen enters the titanium matrix, it occupies interstitial sites in the α-Ti lattice, induces local lattice distortion and increases the resistance to interface migration. During the continuous thermal cycling of WAAM, the formation and growth of α lamellae depend on nucleation, interface migration and elemental diffusion during β-phase decomposition. An appropriate amount of oxygen may suppress the lateral coarsening of α lamellae, thereby reducing the α-lamellar width and increasing the lamellar aspect ratio. Therefore, the α-lamellar refinement observed in the 500 ppm and 1000 ppm specimens is not an isolated phenomenon, but results from the combined effects of oxygen solid solution, enhanced α-phase stability and repeated thermal cycling.

It should be noted that lamellar refinement under controlled oxygen concentrations does not imply severe oxidative damage to the microstructure. Combined with the compositional results discussed above, the internal oxygen contents of the 1 ppm, 500 ppm and 1000 ppm specimens were 0.07 wt.%, 0.10 wt.% and 0.15 wt.%, respectively, all below the ASTM B381 limit. Therefore, within this oxygen concentration range, oxygen mainly participates in microstructural evolution in the form of interstitial solid solution. Its role can be understood as limited solid-solution regulation rather than oxide-induced embrittlement. This point is critical for interpreting the subsequent changes in hardness and tensile properties. Under controlled oxygen concentrations, the increases in strength and hardness mainly arise from oxygen solid-solution strengthening and microstructural refinement, rather than oxide strengthening [28].

In contrast, the microstructure of the specimen fabricated in ambient air was markedly different. In the low-magnification microstructure, the banded structure in the top and middle regions of the specimen was obviously weakened, and α-colony aggregation with different orientations appeared in local regions. The high-magnification SEM results show that the α lamellae in the air-fabricated specimen exhibited more concentrated orientations in some regions, with increased lamellar spacing. White particle-like features were also observed near local grain boundaries in the ambient-air specimen. Combined with the TiO_2_-related diffraction peaks detected by XRD, these features suggest the possible formation of local oxide-related or oxygen-enriched regions under ambient-air exposure. These observations indicate that, when the forming atmosphere changed from a controlled low-oxygen environment to ambient-air exposure, the effect of oxygen was no longer limited to α-lamellar regulation, but was accompanied by severe oxidation-related microstructural degradation.

The abrupt microstructural change in the air-fabricated specimen is closely related to its significantly increased internal oxygen content [10]. The internal oxygen content of this specimen reached 0.36 wt.%, clearly exceeding the standard limit for Ti-6Al-4V. Under this condition, excessive oxygen is difficult to maintain completely as a stable interstitial solute in the titanium matrix. Instead, it may segregate near grain boundaries, lamellar interfaces or remelted interfaces, promoting the formation of oxide-related or oxygen-enriched regions [14]. Owing to the poor deformation compatibility between oxide-related or oxygen-enriched regions and the titanium matrix, these regions may reduce local interfacial continuity and provide preferential sites for crack initiation during subsequent tensile loading. Therefore, the α-colony aggregation, lamellar coarsening and grain-boundary particles observed in the air-fabricated specimen are not isolated microstructural features, but direct manifestations of microstructural embrittlement under severe oxygen contamination.

The microstructural observations indicate that oxygen affects WAAM Ti-6Al-4V in two different regimes. Within the controlled oxygen concentration range, oxygen uptake mainly modifies the α-lamellar morphology while maintaining continuous interlayer bonding and the typical WAAM layered structure. Under ambient-air exposure, excessive oxygen promotes oxide-associated microstructural degradation, as reflected by α-colony aggregation, local lamellar coarsening and grain-boundary particles.

### 3.3. Phase Constitution and Oxide Formation Behavior

**Figure 6:**
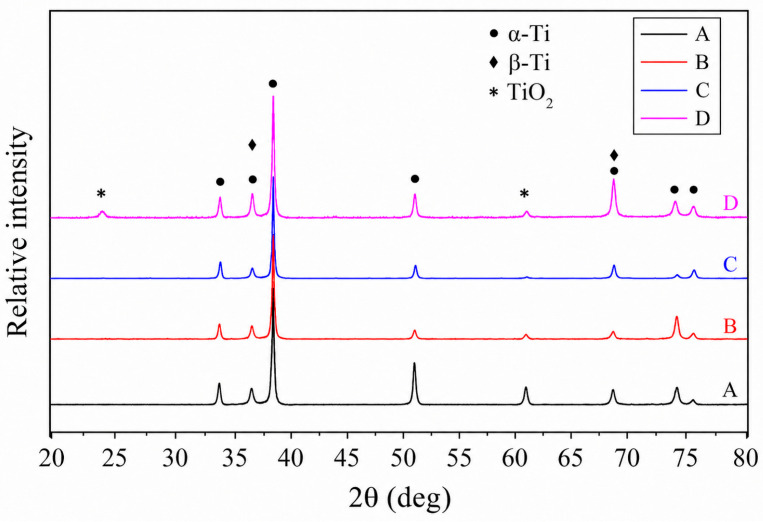
XRD patterns of WAAM Ti-6Al-4V thin-walled specimens fabricated under different ambient oxygen concentrations. Specimens A, B, C and D correspond to 1 ppm, 500 ppm, 1000 ppm and ambient air, respectively.

Figure 6 shows the XRD patterns of the WAAM Ti-6Al-4V specimens fabricated under different oxygen concentrations. The specimens fabricated at 1 ppm, 500 ppm and 1000 ppm were mainly composed of α-Ti and a small amount of β-Ti, and no distinct TiO_2_ diffraction peaks were detected. This indicates that, within the controlled oxygen concentration range, oxygen did not form a detectable amount of crystalline oxide phase. Instead, its influence should be mainly associated with interstitial oxygen in the titanium matrix, possible low-content oxidation products below the XRD detection limit, and changes in α-lamellar morphology. Therefore, for the specimens fabricated at oxygen concentrations from 1 ppm to 1000 ppm, the influence of oxygen on the microstructure and properties should be interpreted mainly in terms of oxygen solid solution, changes in α-lamellar morphology and the surface oxidation-affected layer, rather than being simply attributed to oxide precipitation.

In contrast, TiO_2_-related diffraction peaks appeared in the specimen fabricated in ambient air, indicating that non-controlled high-oxygen exposure had led to the formation of XRD-detectable crystalline oxide phases [29]. The internal oxygen content of this specimen reached 0.36 wt.%, clearly exceeding the standard limit. The particle-like features observed near local grain boundaries in the SEM microstructure are therefore discussed as possible oxide-related or oxygen-enriched features, but they are not used as direct local evidence for TiO_2_ because SEM morphology alone cannot determine their chemical identity. Thus, the TiO_2_-related phase identification in this study is mainly based on the XRD results. This indicates that, when the forming atmosphere changed from a controlled low-oxygen environment to ambient air exposure, the role of oxygen was no longer limited to solid-solution strengthening and microstructural regulation, but further developed into severe oxidation-related microstructural degradation.

### 3.4. Microhardness and Characteristics of the Surface Oxide Layer

**Figure 7:**
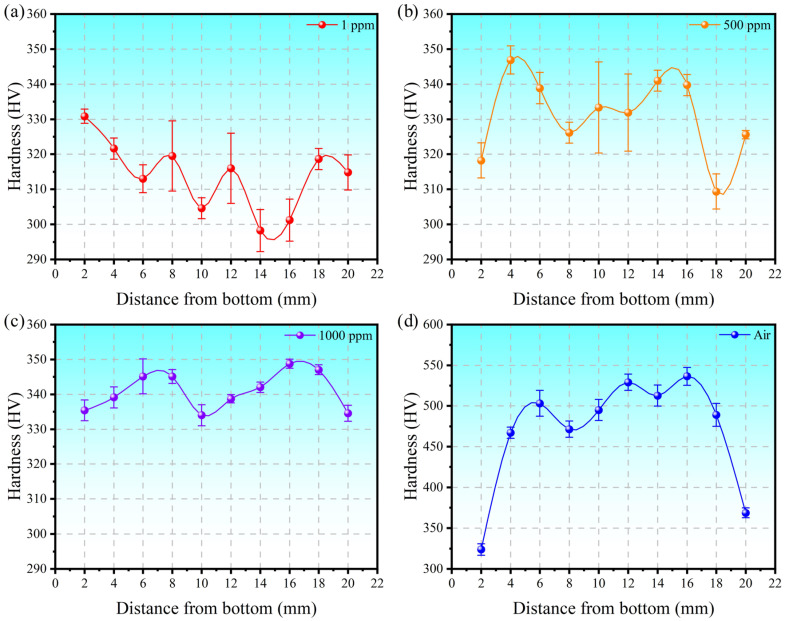
Microhardness distributions along the vertical direction of the cross-sections of WAAM Ti-6Al-4V thin-walled specimens fabricated under different ambient oxygen concentrations. (**a**) 1 ppm, (**b**) 500 ppm, (**c**) 1000 ppm, and (**d**) ambient air.

Figure 7 shows the microhardness distributions along the building direction of the WAAM Ti-6Al-4V thin-walled specimens fabricated under different ambient oxygen concentrations. The hardness of the specimen fabricated at 1 ppm was approximately 320 HV, indicating that the specimen produced under a highly purified atmosphere retained a hardness level typical of WAAM Ti-6Al-4V with a lamellar α + β microstructure (Figure 7a). When the oxygen concentration increased to 500 ppm and 1000 ppm, the hardness increased to approximately 330–350 HV (Figure 7b,c). The ambient-air specimen exhibited much higher and more pronounced hardness values than the specimens fabricated under controlled oxygen concentrations, indicating that severe oxygen exposure produced strong local hardening. However, this hardness increase should be regarded as a local indentation response rather than direct evidence of improved structural integrity.

The hardness increase in the specimens fabricated at 500 ppm and 1000 ppm can be mainly attributed to the combined effects of interstitial oxygen strengthening and α-lamellar refinement. As shown by the chemical composition results, the internal oxygen content increased from 0.07 wt.% at 1 ppm to 0.10 wt.% and 0.15 wt.% at 500 ppm and 1000 ppm, respectively. As these oxygen contents remained below the upper limit specified by ASTM B381-2013, oxygen in these specimens can be regarded mainly as an interstitial solute rather than as a source of severe oxide contamination. Oxygen atoms occupying interstitial sites in the α-Ti lattice induce local lattice distortion and increase the resistance to dislocation motion. Under indentation loading, this effect increases the stress required for local plastic deformation, resulting in higher microhardness [30]. In addition, the refinement of α lamellae observed in the 500 ppm and 1000 ppm specimens can increase the density of α/β interfaces and further contribute to the resistance to localized deformation. Therefore, the hardness increase under controlled oxygen concentrations should be understood as a result of oxygen solid-solution strengthening assisted by microstructural refinement, rather than oxide-particle strengthening.

The hardness distribution along the building direction also reflects the combined influence of thermal cycling and oxygen uptake during layer-by-layer deposition. For the 1 ppm specimen, the hardness fluctuated within a relatively narrow range, indicating that the influence of oxygen uptake was limited under this atmosphere. For the 500 ppm and 1000 ppm specimens, the hardness in the middle and upper regions was generally higher than that in the lower region. This vertical variation suggests that oxygen uptake was not completely uniform throughout the deposited wall, but was affected by the local thermal history and repeated exposure of previously deposited layers. The lower region was deposited earlier and was closer to the substrate, where heat extraction was stronger and the time available for repeated surface oxidation and oxygen accumulation was relatively limited. In contrast, the middle and upper regions experienced repeated thermal exposure, reheating and partial remelting during subsequent deposition. These processes promoted oxygen uptake and redistribution, thereby leading to a relatively higher hardness in these regions. For the ambient-air specimen, the hardness fluctuated more markedly along the building direction than in the specimens fabricated under controlled oxygen concentrations. This fluctuation can be associated with the heterogeneous microstructure produced under severe oxygen exposure, including coarse α colonies, locally concentrated lamellar orientations and weakened layer-band features, as shown in Figure 3 and Figure 4. In addition, TiO_2_-related phases were detected by XRD, and oxide-related or oxygen-enriched regions may have formed locally. These microstructural heterogeneities can lead to local differences in oxygen enrichment, α/β interface density and deformation compatibility within the tested regions, thereby producing a more scattered hardness response.

**Figure 8:**
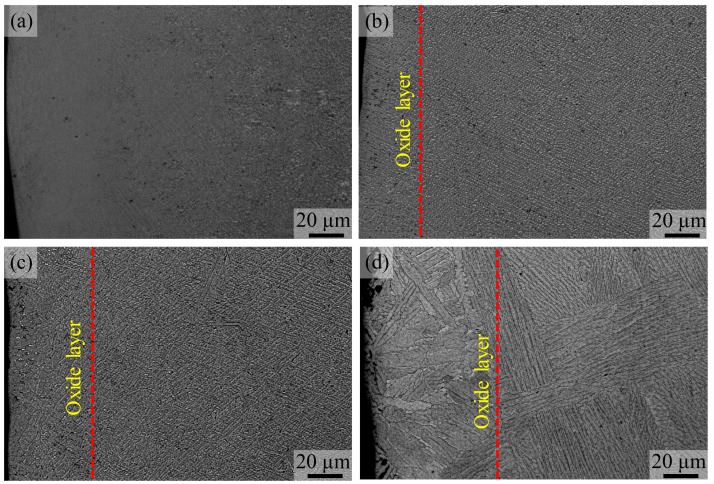
Surface oxide layer morphologies and thicknesses of WAAM Ti-6Al-4V specimens fabricated under different ambient oxygen concentrations. (**a**) 1 ppm, (**b**) 500 ppm, (**c**) 1000 ppm, and (**d**) ambient air.

Figure 8 shows the SEM morphologies near the surface region of the cross-sections of the specimens fabricated under different ambient oxygen concentrations. No obvious oxidation-affected features were observed in the near-surface microstructure of the specimen fabricated at 1 ppm (Figure 8a), whereas oxidation-affected layers of varying degrees appeared in the near-surface regions of the specimens fabricated at 500 ppm, 1000 ppm and ambient air. The measured oxide layer thicknesses were 34.14 ± 3.5 μm, 48.86 ± 4.8 μm and 77.14 ± 7.6 μm for the 500 ppm, 1000 ppm and ambient-air specimens, respectively (Figure 8b–d). These results indicate that the ambient oxygen concentration affects not only the internal oxygen content of the specimens but also the formation and thickening of the surface oxidation-affected layer. As the oxygen concentration in the forming atmosphere increased, the oxidation-affected region expanded progressively from a thin near-surface layer under controlled atmospheres to a much thicker degraded surface region under ambient-air exposure.

The development of the surface oxidation-affected layer can be associated with the high chemical affinity between titanium and oxygen at elevated temperature. During deposition, the surface of each newly formed layer is exposed to the surrounding atmosphere while it remains at high temperature. Oxygen can react with titanium to form an oxide film or oxygen-enriched surface layer. During the deposition of subsequent layers, part of this surface layer may be reheated, partially remelted or entrained into the newly formed molten pool. Therefore, the final oxygen-related response of the deposited wall is not only determined by the nominal oxygen concentration in the atmosphere, but also by the repeated oxidation, reheating and remelting history during multilayer deposition.

From a kinetic viewpoint, oxygen pickup during WAAM can be further understood as a cumulative process involving surface reaction, thermally activated diffusion and local remelting. During multilayer deposition, previously deposited regions can experience repeated reheating cycles, and subsequent layers may partially remelt the oxidation-affected surface region. This thermal-cycle characteristic has also been reported in previous studies on GTAW-WAAM Ti-6Al-4V. During reheating, oxygen can diffuse from the oxygen-enriched near-surface region into the titanium matrix, and the diffusion coefficient increases strongly with temperature following an Arrhenius-type dependence. Although each high-temperature exposure is limited in duration, repeated reheating increases the effective time for oxygen redistribution. In addition, local remelting may entrain part of the oxidation-affected surface layer into the molten pool, further contributing to internal oxygen pickup. Under controlled oxygen atmospheres, the limited oxygen supply mainly leads to near-surface oxygen enrichment and interstitial oxygen solid solution. Under ambient-air exposure, the much higher oxygen supply thickens the oxidation-affected layer and promotes oxygen transport into the interior during repeated reheating and remelting, eventually resulting in severe oxygen contamination and oxidation-related microstructural degradation.

Although oxide layers were observed in the 500 ppm and 1000 ppm specimens, their influence should be distinguished from the severe oxidation observed in the ambient-air specimen. For the 500 ppm and 1000 ppm specimens, the near-surface region is described as an oxidation-affected layer to reflect the combined influence of oxygen uptake and surface oxidation, rather than a continuous crystalline TiO_2_ layer. Considering the absence of distinct TiO_2_ diffraction peaks and the increase in near-surface hardness, this region may be more appropriately interpreted as an oxygen-enriched or α-case-like layer under the present controlled oxygen atmospheres. In these two controlled-atmosphere specimens, the internal oxygen contents were still below the ASTM limit, and no obvious TiO_2_ diffraction peaks were detected by XRD. This indicates that the oxidation-affected layer did not develop into extensive internal oxide contamination under these controlled conditions. Therefore, the main mechanical effect of oxygen in these specimens remained solid-solution strengthening and near-surface hardening. By contrast, the ambient-air specimen showed a much thicker oxidation-affected layer, an internal oxygen content of 0.36 wt.% and TiO_2_-related diffraction peaks. These features indicate that ambient-air exposure caused a transition from controlled surface oxidation to severe oxygen contamination, involving both surface-layer degradation and internal oxide formation.

**Figure 9:**
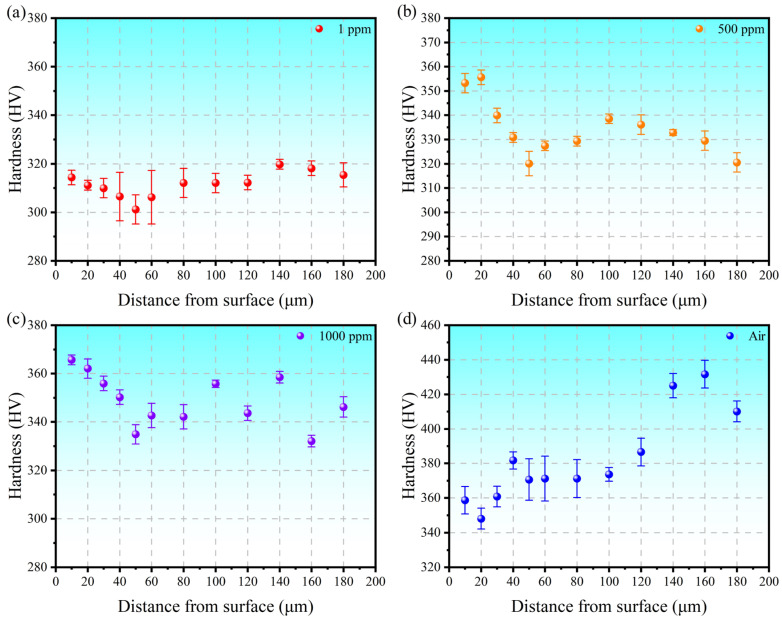
Microhardness gradient distributions from the surface to the interior of WAAM Ti-6Al-4V specimens fabricated under different ambient oxygen concentrations. (**a**) 1 ppm, (**b**) 500 ppm, (**c**) 1000 ppm, and (**d**) ambient air.

Figure 9 further shows the hardness gradient from the surface to the interior of the specimens. The 1 ppm specimen exhibited a relatively uniform hardness distribution from the surface to the interior, fluctuating around 320 HV (Figure 9a), which is consistent with the absence of a clear oxidation-affected layer. For the 500 ppm and 1000 ppm specimens, the near-surface hardness was clearly higher than the internal hardness, by approximately 20 HV (Figure 9b,c). This near-surface hardening is consistent with the formation of oxygen-enriched surface regions under controlled oxygen atmospheres. In contrast, the specimen fabricated in ambient air showed a different gradient feature, where the near-surface hardness was not higher than the internal hardness and was even lower than that of the inner region (Figure 9d). This phenomenon indicates that excessive oxidation does not necessarily lead to a higher near-surface hardness. In the ambient-air specimen, the near-surface region contained a much thicker oxidation-affected layer and more pronounced oxidation-related microstructural heterogeneity, as supported by Figure 8 and the TiO_2_-related XRD peaks. These features may reduce local deformation compatibility and load-bearing continuity during indentation, resulting in a lower near-surface hardness than that of the oxygen-enriched interior. Meanwhile, the inner region can still retain a relatively high hardness because of oxygen enrichment in the matrix. Therefore, the ambient-air specimen exhibited a combined feature of internal hardening and surface deterioration.

Microhardness mainly reflects the local resistance to indentation deformation and cannot fully represent the overall load-bearing behavior under tensile loading [31]. This is particularly important for the ambient-air specimen, in which high local hardness coexisted with oxide layer thickening, oxide formation and surface microstructural degradation. Therefore, the hardness results should be interpreted together with the tensile properties and fracture morphologies discussed below.

### 3.5. Tensile Properties and Fracture Behavior

**Figure 10:**
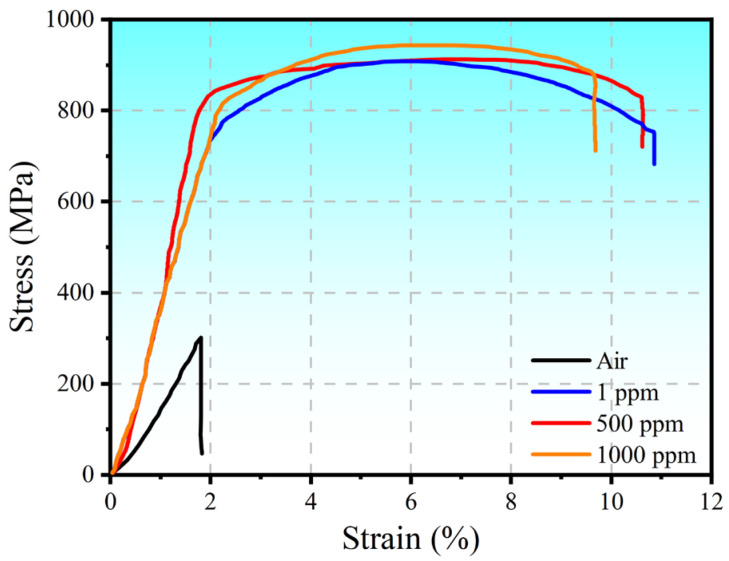
Room-temperature tensile stress–strain curves of WAAM Ti-6Al-4V specimens fabricated under different ambient oxygen concentrations.

**Figure 11:**
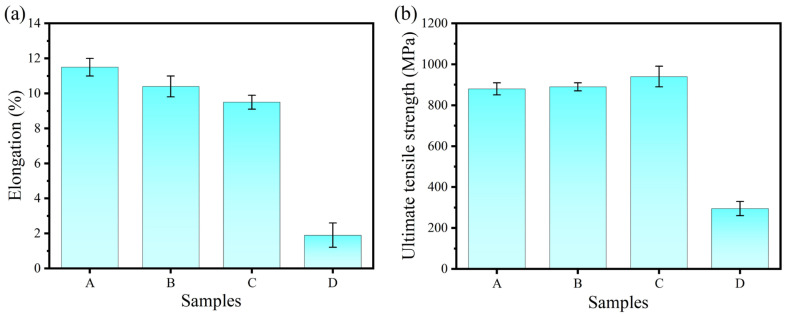
Statistical results of tensile properties of WAAM Ti-6Al-4V specimens fabricated under different ambient oxygen concentrations. (**a**) Elongation and (**b**) ultimate tensile strength.

Figure 10 shows the room-temperature tensile stress–strain curves of the WAAM Ti-6Al-4V specimens fabricated under different ambient oxygen concentrations. The corresponding statistical tensile properties are summarized in Figure 11, where the data are presented as mean values with standard deviations from three tensile specimens for each condition. The tensile properties of the specimens exhibited a distinct staged variation with increasing ambient oxygen concentration. At an oxygen concentration of 1 ppm, the specimen showed a tensile strength of 880 ± 30 MPa and an elongation of 11.5 ± 0.5%. Within the controlled oxygen concentration range from 1 ppm to 1000 ppm, the tensile strength remained at a comparable level at 500 ppm and then increased at 1000 ppm, whereas the elongation gradually decreased with increasing ambient oxygen concentration. At 500 ppm, the tensile strength and elongation were 870 ± 20 MPa and 10.4 ± 0.6%, respectively. When the oxygen concentration increased to 1000 ppm, the tensile strength increased to 940 ± 50 MPa, while the elongation decreased to 9.5 ± 0.4%. In contrast, the tensile properties of the specimen fabricated in ambient air deteriorated significantly, with the tensile strength and elongation decreasing to only 295 ± 35 MPa and 1.9 ± 0.7%, respectively. These results indicate that the effect of ambient oxygen concentration on the tensile properties of WAAM Ti-6Al-4V is not a simple monotonic strengthening process, but instead exhibits a clear critical transition. Within the controlled oxygen concentration range, the increase in oxygen content mainly leads to strength retention or enhancement accompanied by ductility loss. In ambient air, however, excessive oxygen exposure causes a sharp simultaneous decrease in both strength and elongation. Therefore, the specimens fabricated at 1 ppm to 1000 ppm and the specimen fabricated in ambient air should be interpreted separately in terms of limited oxygen solid-solution strengthening and embrittlement induced by excessive oxygen contamination.

As the internal oxygen content increased from 0.07 wt.% to 0.15 wt.%, interstitial oxygen atoms introduced lattice distortion in the α-Ti matrix and increased the resistance to dislocation motion. Meanwhile, the α-lamellar structure became finer at 500 ppm and 1000 ppm, which further increased the number of interfaces that can hinder local deformation. These two factors jointly contributed to the increase in tensile strength from 880 ± 30 MPa to 940 ± 50 MPa. Therefore, within the oxygen concentration range from 1 ppm to 1000 ppm, the strengthening of WAAM Ti-6Al-4V mainly resulted from oxygen solid-solution strengthening and α-lamellar refinement [32].

However, the increase in strength was accompanied by a decrease in elongation. This strength–ductility trade-off is reasonable for Ti-6Al-4V containing increased interstitial oxygen. Oxygen improves the resistance to dislocation motion, but at the same time reduces the ability of the matrix to accommodate plastic deformation. As a result, the material requires a higher stress to deform, but its capacity for uniform deformation before fracture becomes lower. The 1000 ppm specimen still maintained an elongation of 9.5%, and no obvious TiO_2_ diffraction peaks or severe interlayer defects were detected. Therefore, the reduction in elongation under this condition should not be regarded as severe embrittlement. Instead, it represents a moderate ductility loss caused by controlled oxygen uptake and matrix strengthening.

The tensile behavior of the specimen fabricated in ambient air was markedly different. Its tensile strength sharply decreased from 880–940 MPa for the specimens fabricated under controlled oxygen concentrations to 295 MPa, and its elongation decreased to 1.9%. This abrupt decrease should not be interpreted as a simple linear effect of the oxygen-content increase from 0.15 wt.% to 0.36 wt.%; rather, the ambient-air specimen should be considered as a different degradation regime, in which excessive oxygen exposure changes the dominant mechanical response from matrix strengthening to oxidation-related damage and brittle fracture. This simultaneous decrease in strength and elongation indicates that the air-fabricated specimen did not undergo “further strengthening accompanied by ductility loss”, but instead suffered severe microstructural damage and brittle failure. Combined with the compositional and phase results discussed above, the internal oxygen content of the air-fabricated specimen reached 0.36 wt.%, clearly exceeding the standard limit, and TiO_2_-related diffraction peaks were detected. Moreover, the ambient-air specimen contained a much thicker oxidation-affected layer, coarse α colonies, locally concentrated lamellar orientations and oxide-related or oxygen-enriched regions. These features can reduce microstructural continuity and interfacial reliability, thereby increasing crack-initiation sensitivity and accelerating crack propagation during tensile loading. This indicates that excessive oxygen no longer affected the matrix only in the form of solid solution, but further caused oxidation-related microstructural degradation. Therefore, the severe deterioration in tensile properties of the ambient-air specimen should be attributed to the combined effects of excessive oxygen uptake, oxidation-related microstructural degradation and brittle fracture behavior, rather than to oxygen solid-solution strengthening alone.

This result also shows that microhardness and tensile properties reflect different aspects of mechanical behavior. Microhardness mainly characterizes local resistance to indentation deformation, whereas tensile properties are controlled by the combined effects of matrix strength, defect sensitivity, interfacial bonding and fracture resistance. For the specimens fabricated at 1 ppm to 1000 ppm, the increase in hardness corresponded reasonably with the increase in tensile strength because the microstructure remained continuous and no severe oxide-induced defects were introduced. For the ambient-air specimen, however, the local hardening caused by oxygen enrichment could not compensate for the deterioration of microstructural continuity and interfacial reliability. Therefore, the hardness increase in the ambient-air specimen should not be interpreted as an improvement in overall mechanical performance.

From the perspective of process control, the specimens fabricated within the range from 1 ppm to 1000 ppm exhibited an increase in strength and a moderate decrease in elongation with increasing oxygen concentration, indicating that, under the processing conditions used in this study, oxygen concentrations below 1000 ppm still fall within an acceptable range. In contrast, ambient air represents a non-controlled high-oxygen exposure condition, which leads to excessive internal oxygen content and severe deterioration in tensile properties. Therefore, atmosphere control for WAAM Ti-6Al-4V should avoid air exposure and severe oxygen contamination, but it does not necessarily require the oxygen concentration to be reduced indefinitely. For the deposition parameters and thin-wall specimen dimensions used in this study, an oxygen concentration below 1000 ppm can be regarded as a meaningful reference range for atmosphere control.

**Figure 12:**
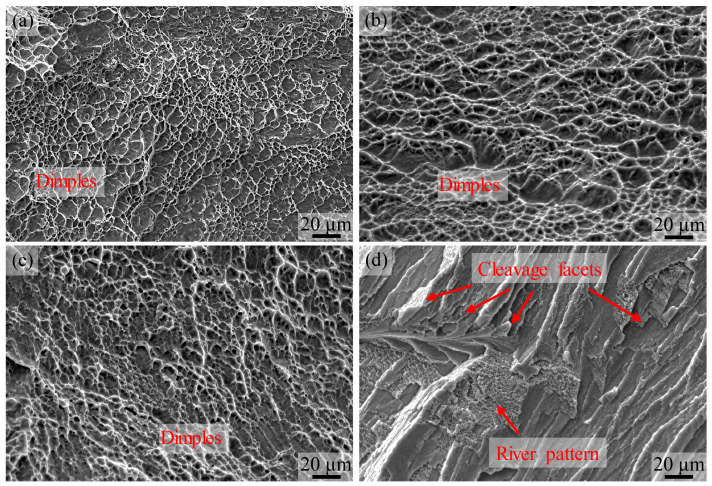
Fracture morphologies of WAAM Ti-6Al-4V tensile specimens fabricated under different ambient oxygen concentrations. (**a**) 1 ppm, (**b**) 500 ppm, (**c**) 1000 ppm, and (**d**) ambient air.

Figure 12 shows the fracture morphologies of the tensile specimens fabricated under different ambient oxygen concentrations. The specimens fabricated at 1 ppm, 500 ppm and 1000 ppm retained ductile fracture features to varying degrees, as evidenced by the dimpled fracture surfaces shown in Figure 12a–c. With increasing oxygen content, the plastic deformation features became less pronounced, consistent with the reduction in elongation. In contrast, the ambient-air specimen exhibited brittle fracture features, including cleavage facets and river patterns (Figure 12d). Combined with the tensile results, it can be seen that the fracture behaviors of the specimens fabricated under controlled oxygen concentrations and that fabricated in ambient air were significantly different. Although the 1 ppm, 500 ppm and 1000 ppm specimens exhibited reduced elongation with increasing oxygen concentration, they still maintained relatively high tensile strength overall, indicating that their fracture processes were still governed by the plastic deformation capability of the matrix. With increasing oxygen content, the plastic deformation features on the fracture surfaces may become weaker, indicating that oxygen solid-solution strengthening increased the deformation resistance while reducing the plastic energy dissipation capacity prior to crack propagation. This is consistent with the tensile-property trend of increased strength and reduced elongation.

In contrast, the fracture surface of the ambient-air specimen exhibited typical brittle features, including relatively flat facets and cleavage-like morphology. These fracture characteristics are consistent with the severe reduction in elongation to 1.9%. The brittle fracture behavior is closely related to the excessive oxygen uptake and TiO_2_-related oxide formation discussed above. Oxide-enriched regions and weakened interfaces can serve as preferential sites for crack initiation, while the reduced plastic deformation capacity of the oxygen-contaminated matrix accelerates crack propagation. Therefore, the fracture mode changed from ductile fracture under controlled oxygen concentrations to brittle fracture under ambient-air exposure.

This result also explains why the specimen fabricated in ambient air exhibited relatively high hardness but significantly degraded tensile properties. Microhardness mainly reflects the ability of a local region to resist indentation deformation, whereas tensile properties depend simultaneously on matrix strength, microstructural continuity, interfacial bonding state and defect sensitivity. In the air-fabricated specimen, oxygen solid solution and surface oxidation increased the local hardness, but TiO_2_ formation, oxide layer thickening and interfacial weakening caused by excessive oxygen significantly reduced the overall load-bearing capacity under tensile loading. Therefore, an increase in hardness cannot be directly equated with an improvement in overall mechanical properties. For specimens exposed to high oxygen levels, the oxide-induced embrittlement effect had already exceeded the beneficial effect of solid-solution strengthening.

Based on the tensile properties and fracture morphologies, the mechanical response of WAAM Ti-6Al-4V under different oxygen concentration conditions can be summarized into two stages. The first stage is the controlled oxygen solid-solution strengthening stage, corresponding to the range from 1 ppm to 1000 ppm. In this stage, the increase in oxygen content enhanced the resistance to dislocation motion and, together with the refinement of α lamellae, promoted an increase in tensile strength. However, the ability to coordinate plastic deformation was reduced, leading to decreased elongation. The second stage is the excessive oxygen contamination-induced embrittlement stage, represented by ambient air. In this stage, the internal oxygen content clearly exceeded the standard limit, TiO_2_-related oxides formed, and grain-boundary and interfacial bonding were weakened. As a result, the dominant mechanism changed from solid-solution strengthening to oxide-induced embrittlement, ultimately causing a sharp simultaneous decrease in strength and elongation.

From the viewpoint of atmosphere control, these results suggest that the key issue in GTAW-WAAM Ti-6Al-4V is not simply to minimize the oxygen concentration without limit, but to prevent the transition from controlled oxygen solid-solution strengthening to excessive oxygen contamination. Under the deposition parameters and specimen geometry used in this study, oxygen concentrations up to 1000 ppm still produced specimens with acceptable internal oxygen contents, continuous microstructures and relatively high tensile properties. It should be emphasized that this value should be regarded as a process-specific reference under the present thin-wall geometry, heat input and deposition conditions, rather than as a universal oxygen-concentration limit for WAAM Ti-6Al-4V. Changes in wall thickness, number of deposited layers, heat input or thermal exposure history may require more restrictive atmosphere-control conditions. However, once effective shielding was lost and the specimen was exposed to ambient air, severe oxidation and embrittlement occurred. Therefore, maintaining a controlled low-oxygen atmosphere is essential for balancing microstructural stability, mechanical reliability and practical atmosphere-control requirements in WAAM Ti-6Al-4V components.

## 4. Conclusions

This study investigated the effects of different ambient oxygen concentrations on oxygen uptake, microstructural evolution and mechanical properties of GTAW-WAAM Ti-6Al-4V thin-walled specimens. The main conclusions are as follows:

(1) As the ambient oxygen concentration increased from 1 ppm to 1000 ppm, the degree of surface oxidation gradually increased, and the surface color changed from silvery white to yellow, blue-gray and locally purple. The internal oxygen contents of the 1 ppm, 500 ppm and 1000 ppm specimens were 0.07 wt.%, 0.10 wt.% and 0.15 wt.%, respectively, all below the limit specified by ASTM B381-2013. In contrast, the oxygen content of the specimen fabricated in ambient air increased to 0.36 wt.%, indicating severe oxygen contamination.
(2) Controlled oxygen uptake below 1000 ppm did not significantly damage the interlayer bonding of the WAAM specimens. The specimens were still mainly composed of α-Ti and a small amount of β-Ti, and exhibited a refinement tendency of α lamellae. In contrast, α-colony aggregation, local lamellar coarsening and TiO_2_-related oxides appeared in the specimen fabricated in ambient air, indicating that excessive oxygen exposure induced microstructural deterioration.
(3) Increasing ambient oxygen concentration increased the microhardness and surface oxide layer thickness of the specimens. The hardness of the 1 ppm specimen was approximately 320 HV, while that of the 500 ppm and 1000 ppm specimens increased to 330–350 HV. The oxide layer thicknesses of the 500 ppm, 1000 ppm and ambient-air specimens were approximately 30 μm, 50 μm and 90 μm, respectively. Although the specimen fabricated in ambient air exhibited relatively high hardness, it was accompanied by surface microstructural damage and oxide formation.
(4) The tensile properties showed a staged response to ambient oxygen concentration. Under the present GTAW-WAAM thin-wall deposition conditions, increasing the oxygen concentration from 1 ppm to 1000 ppm increased the tensile strength from 880 MPa to 940 MPa, while reducing the elongation from 11.5% to 9.5%, indicating a strength–ductility trade-off caused by limited oxygen solid-solution strengthening. In contrast, the specimen fabricated in ambient air showed a sharp decrease in tensile strength and elongation to 295 MPa and 1.9%, respectively, with brittle fracture characteristics. These results indicate that atmosphere control for WAAM Ti-6Al-4V should prevent the transition from controlled oxygen strengthening to excessive oxygen-induced embrittlement.

## Figures and Tables

**Table 1:** Representative bulk chemical compositions of WAAM Ti-6Al-4V specimens fabricated under different atmosphere conditions.

Sample	O (wt.%)	Al (wt.%)	V (wt.%)
A	0.07	5.78	3.77
B	0.10	5.87	3.73
C	0.15	5.88	3.79
D	0.36	5.97	3.84

## Data Availability

The original contributions presented in this study are included in the article. Further inquiries can be directed to the corresponding authors.

## References

[1] Hasib M.T., Ostergaard H.E., Li X., Kruzic J.J. (2021). Fatigue crack growth behavior of laser powder bed fusion additive manufactured Ti-6Al-4V: Roles of post heat treatment and build orientation. Int. J. Fatigue.

[2] Aufa A., Hassan M.Z., Ismail Z. (2022). Recent advances in Ti-6Al-4V additively manufactured by selective laser melting for biomedical implants: Prospect development. J. Alloys Compd..

[3] Li M., Zhang Y., Liu B., Shi J., Yu K., Li J. (2026). Mechanism of heat treatment regulation on the formation of Ti-6Al-4V oxide film in additive manufacturing and its corrosion resistance: Synergistic effect of matrix structure and residual stress. Corros. Sci..

[4] Lin Z., Song K., Yu X. (2021). A review on wire and arc additive manufacturing of titanium alloy. J. Manuf. Process..

[5] Srivastava M., Jayakumar V., Udayan Y., Sathishkumar M., Muthu M.S., Gautam P., Nag A. (2024). Additive manufacturing of Titanium alloy for aerospace applications: Insights into the process, microstructure, and mechanical properties. Appl. Mater. Today.

[6] Hao Y., Rufeng H., Huajun C., Menglin L., Jin Z. (2021). Research progress and prospects of CMT-based wire arc additive manufacturing technology for titanium alloys. China Surf. Eng..

[7] Gain S., Veeman D. (2025). A review on advances and challenges in wire arc additive manufacturing: Process parameters, microstructural evolution and material performance across alloys. J. Alloys Compd..

[8] Badhan S.K., Raja C.P., Chakraborty A., Mensah R.A. (2026). Bridging processing and Performance in Ti-6Al-4V: WAAM parameters and Wear Behaviour across Traditional and Additive Manufacturing Routes. Front. Mater..

[9] Bermingham M., Thomson-Larkins J., St John D., Dargusch M. (2018). Sensitivity of Ti-6Al-4V components to oxidation during out of chamber Wire+ Arc Additive Manufacturing. J. Mater. Process. Technol..

[10] Halisch C., Milcke B., Radel T., Rentsch R., Seefeld T. (2023). Influence of oxygen content in the shielding gas chamber on mechanical properties and macroscopic structure of Ti-6Al-4V during wire arc additive manufacturing. Int. J. Adv. Manuf. Technol..

[11] Chong Y., Tsuru T., Gholizadeh R., Minor A.M., Tsuji N. (2025). Mechanistic origin of oxygen-induced twin suppression in titanium. Acta Mater..

[12] Yu A., Pan Y., Liu Y., Zheng T., Wen Y., Wu Y., Lu X. (2025). Harnessing oxygen-driven phase engineering for a strong and ductile duplex titanium alloy. Adv. Compos. Hybrid Mater..

[13] Mao Y., Zhao Q., Geng J., Zhang R., Guo P., Wang N., Chen Y., Zhao Y. (2025). Overcoming strength-ductility trade-off in titanium alloy by tailoring and regulating local-range ordered oxygen structure. Acta Mater..

[14] Duan H., Zhang H., Cheng X., Mu X., Fan Q., Zhang Y., Xiong N., Feng K., Wang Y., Li X. (2025). Achieving high oxygen tolerance in Ti6Al4V: Copper-oxygen co-doping strategy for ultrahigh strength-ductility balance. Mater. Des..

[15] Chong Y., Gholizadeh R., Tsuru T., Zhang R., Inoue K., Gao W., Godfrey A., Mitsuhara M., Morris J., Minor A.M. (2023). Grain refinement in titanium prevents low temperature oxygen embrittlement. Nat. Commun..

[16] Ren Y., Xu J., Wei Y., Liu Y., Zhu J., Liu S. (2025). Effect of interstitial oxygen on the microstructure and mechanical properties of titanium alloys: A review. Crystals.

[17] Mansor M.S.M., Raja S., Yusof F., Muhamad M.R., Manurung Y.H., Adenan M.S., Hussein N.I.S., Ren J. (2024). Integrated approach to Wire Arc Additive Manufacturing (WAAM) optimization: Harnessing the synergy of process parameters and deposition strategies. J. Mater. Res. Technol..

[18] Zhou Y., Qin G., Li L., Lu X., Jing R., Xing X., Yang Q. (2020). Formability, microstructure and mechanical properties of Ti-6Al-4V deposited by wire and arc additive manufacturing with different deposition paths. Mater. Sci. Eng. A.

[19] Halder R., Pistorius P.C., Blazanin S., Sardey R.P., Quintana M.J., Pierson E.A., Verma A.K., Collins P.C., Rollett A.D. (2024). The effect of interlayer delay on the heat accumulation, microstructures, and properties in laser hot wire directed energy deposition of Ti-6Al-4V single-wall. Materials.

[20] Cai Y., Peng Z., Chen J., Chen H., Xiong J. (2024). Grain refinement and anisotropy improvement of arc-directed energy deposited Ti–6Al–4V with oscillating laser. Mater. Sci. Eng. A.

[21] Syed A.K., Zhang X., Davis A.E., Kennedy J.R., Martina F., Ding J., Williams S., Prangnell P.B. (2021). Effect of deposition strategies on fatigue crack growth behaviour of wire+ arc additive manufactured titanium alloy Ti–6Al–4V. Mater. Sci. Eng. A.

[22] Kishor G., Mugada K.K., Mahto R.P. (2026). Wire arc additive manufacturing of titanium alloys for enhancing mechanical properties and grain-refinement. Met. Mater. Int..

[23] Wu B., Qiu Z., Dong B., Wexler D., Pan Z., Carpenter K., Corradi D.R., Li H. (2022). Effects of synchronized magnetic arc oscillation on microstructure, texture, grain boundary and mechanical properties of wire arc additively manufactured Ti6Al4V alloy. Addit. Manuf..

[24] Thomson-Larkins J. (2017). Sensitivity of Ti-6Al-4V Components to Oxidation During out of Chamber Wire Arc Additive Layer Manufacturing (WAALM). Bachelor’s Thesis.

[25] Wang H., Chao Q., Cui X., Chen Z., Breen A., Cabral M., Haghdadi N., Huang Q., Niu R., Chen H. (2022). Introducing C phase in additively manufactured Ti-6Al-4V: A new oxygen-stabilized face-centred cubic solid solution with improved mechanical properties. Mater. Today.

[26] Choi Y., Jeong C. (2025). Correlation of oxide film thickness with interference coloration and corrosion resistance in anodized titanium. Electrochim. Acta.

[27] (2013). Standard Specification for Titanium and Titanium Alloy Forgings.

[28] Nurly H.F., Ren D., Cai Y., Ji H., Wang H., Huang A., Yang R. (2024). Effects of oxygen on microstructure and mechanical properties of selective laser melted Ti–6Al–4V annealed at different temperatures. Mater. Sci. Eng. A.

[29] Singla J., Kumar N., Bansal A. (2025). Manufacturing of Ti6Al4V alloy using argon chamber assisted WAAM-CMT to avoid oxidation and analyses of mechanical and metallurgical properties. J. Alloys Compd..

[30] Wang X.-Q., Zhang Y.-S., Han W.-Z. (2022). Design of high strength and wear-resistance β-Ti alloy via oxygen-charging. Acta Mater..

[31] Karolewska K., Wirwicki M., Ligaj B. (2025). Evaluation of a Method for Determining Material Strength Based on Hardness Measurements: A Case Study of the Ti6Al4V Alloy. Materials.

[32] Kondoh K., Ichikawa E., Issariyapat A., Shitara K., Umeda J., Chen B., Li S. (2020). Tensile property enhancement by oxygen solutes in selectively laser melted titanium materials fabricated from pre-mixed pure Ti and TiO_2_ powder. Mater. Sci. Eng. A.

